# Deep Learning for Efficient and Optimal Motion Planning for AUVs with Disturbances

**DOI:** 10.3390/s21155011

**Published:** 2021-07-23

**Authors:** Juan Parras, Patricia A. Apellániz, Santiago Zazo

**Affiliations:** Information Processing and Telecommunications Center, E.T.S. Ingenieros de Telecomunicación, Universidad Politécnica de Madrid, 28040 Madrid, Spain; patricia.alonsod@upm.es (P.A.A.); santiago.zazo@upm.es (S.Z.)

**Keywords:** optimal control, Hamilton–Jacobi–Bellman equation, autonomous underwater vehicle, Deep Galerkin Method

## Abstract

We use the recent advances in Deep Learning to solve an underwater motion planning problem by making use of optimal control tools—namely, we propose using the Deep Galerkin Method (DGM) to approximate the Hamilton–Jacobi–Bellman PDE that can be used to solve continuous time and state optimal control problems. In order to make our approach more realistic, we consider that there are disturbances in the underwater medium that affect the trajectory of the autonomous vehicle. After adapting DGM by making use of a surrogate approach, our results show that our method is able to efficiently solve the proposed problem, providing large improvements over a baseline control in terms of costs, especially in the case in which the disturbances effects are more significant.

## 1. Introduction

The problem of motion planning for Autonomous Underwater Vehicles (AUVs) is to choose the best trajectory that satisfies a set of constraints, such as the maximum acceleration and velocity of the AUV. Among all trajectories that satisfy these constrains, the best trajectory is the one that optimizes a certain metric, such as time to reach a certain target or computation time. Traditionally, there have been two ways to address the problem of motion planning for AUVs: the field of robotics, which pays attention specially to computational issues and real-time control, and the field of optimal control, which emphasizes the optimization of the trajectory regarding a certain performance measure [1].

Even though the optimal control approach is very attractive, as it is able to return the best trajectory that fulfills the constrains regarding the performance metric chosen, it suffers from the so called “curse of dimensionality”: as the dimensionality of the problem grows (i.e., the number of state variables of the AUV), the computational resources required to use optimal control tools become too high. Even though there are exceptional cases in which the solution is known, as the case of linear quadratic problems [2], the use of optimal control tools in general leads to an intractable problem. As optimal control applies to many problems of interest in different fields, a lot of efforts have been addressed to find efficient ways to solve, in an exact or approximate way, optimal control problems, such as [3,4,5,6,7,8]. Some of these works are still unable to scale up to practical problems, and others require a specific structure of the problem. However, the recent advances in the deep learning field have brought a novel approach to solve optimal control problems, based on the Deep Galerkin Method (DGM, [9]) that can be used to solve optimal control problems without being subject to the “curse of dimensionality” [10] and is becoming popular due to its competitive results [11,12,13].

In this work, we make use of DGM to develop a method that is able to address motion planning problems for AUVs using optimal control tools. The use of neural networks to address motion planning problems for ocean navigation is not new, as [14] already makes use of such tools to approximate the solution of a Kalman filter for a surface ship navigation problem. In [15], the authors use optimal control tools for underwater navigation, but they have to use a restricted mesh of discrete states to avoid the curse of dimensionality (as in [16] for surface vehicles). A recent work close to ours is [17], where the authors use optimal control tools for mine detection missions. They use autonomous vehicles that move in the ocean surface, and seek to obtain optimal trajectories in the sense that they maximize the mine detection probability using different detection sensor models. However, they use coverage path planning, which consists of solving the optimal control problem in a discrete environment, and then check whether the discrete solution can be applied to a continuous system. A similar procedure is described in [18], where the authors first find a discrete solution and then interpolate it. In contrast to these works, which rely on discretization of the states, we solve the problem in the continuous domain, and also use a different target function, as we intend to minimize the time to reach a certain target. Another popular approach is based on using Model Predictive Control (MPC), which can be used to approximate nonlinear control problems in an online or offline fashion [19,20,21,22]. MPC differs from our approach in that we obtain the optimal solution by obtaining first the value function and also our approach may deal with continuous time without having to use discretization.

A problem that arises in the underwater medium is localization: as GPS does not reach the ocean depths, it is necessary to develop other location systems. This topic is subject to intense research, as shown in [23], as we need accurate localization mechanisms to implement optimal trajectories. However, this paper is focused on obtaining optimal trajectories, and hence, we will consider that there is a localization mechanism in the AUV such that its position and velocities are known (i.e., fully observable).

The main contributions of our work are the following:

1. We solve a motion planning problem for an AUV using computationally efficient tools, namely, DGM, which solves a continuous time and states nonlinear optimal control problem. This tool will prove efficient computationally, and departs from numerous approaches that rely on state discretization;
2. The optimal control problem we focus on consists of reaching a target position as soon as possible. As this problem cannot be solved directly using DGM, as DGM required a fixed time horizon, we develop a surrogate problem that can be solved using DGM and returns an equivalent control to our original control problem. This supposes an advance, as it extends the range of uses of DGM to problems with an unknown time horizon;
3. We take into account the effect of several disturbances in the computation of our optimal trajectory. The presence of disturbances is frequent in the ocean, where currents and swirls affect the motion of the AUV. We model some disturbances and show that DGM is able to obtain optimal trajectories that take into account the effect of the disturbance.

In short, we make use of the recent advances in deep learning to efficiently solve the optimal control problem applied to AUV motion planning in the presence of disturbances. The rest of this paper goes as follows: Section 2 presents our setup and the disturbances that we use in this work. Then, Section 3 presents the optimal control tools that we use, and shows how it is possible to use DGM to solve a surrogate optimal control problem for motion planning efficiently. Our ideas are validated via simulations in Section 4, and finally, we draw some conclusions in Section 5.

## 2. Setup Description

### 2.1. Underwater Navigation Model

We consider an AUV that moves in the plane (i.e., constant depth), where the AUV position is the vector (x,y), its velocity is $\left(v_{x},v_{y}\right)$, and we consider that the control variable is the acceleration angle θ. If we assume that the disturbances affect the acceleration and are modeled by the vector $\left(p_{x},p_{y}\right)$, and if we model the friction by a term that depends on the velocity and a parameter $k_{f}$, the motion is controlled by the next Ordinary Differential Equation (ODE) system:

(1) $$\begin{array}{cc} \dot{x}\left(t\right) & =v_{x}\left(t\right)\\ \dot{y}\left(t\right) & =v_{y}\left(t\right)\\ \dot{v_{x}}\left(t\right) & =\cos\left(\theta \left(t\right)\right)+p_{x}\left(x\left(t\right),y\left(t\right)\right)-k_{f}\cdot v_{x}\left(t\right)\\ \dot{v_{y}}\left(t\right) & =\sin\left(\theta \left(t\right)\right)+p_{y}\left(x\left(t\right),y\left(t\right)\right)-k_{f}\cdot v_{y}\left(t\right) \end{array}$$

where $\dot{x}$ represents the derivative with respect to time of the variable *x*. Note that $k_{f}$ limits the maximum velocity that the AUV can reach (see the Isotropic Rocket problem [24]). Additionally, it can be observed that we consider that the disturbances depend only on the AUV position. Finally, note that we choose to solve a planar problem because we can plot the results in a meaningful way, but all our developments can be easily extended to deal with 3-D models as well. It is important to note that the procedure we describe in this work could be applied to a different modeling of the problem; for instance, if the friction came from pressure drag, it would be proportional to the square of velocity.

### 2.2. Disturbance Models

We model three disturbances that appear in the underwater environment: swirls, currents and constant fields. The constant field is a fixed disturbance model that uses two scalar parameters a∈R and α∈[0,2π), where the absolute value of *a* controls the strength of the field and α its orientation as:

(2) $$\begin{array}{cc} p_{x} & =a\cdot \cos(\alpha )\\ p_{y} & =a\cdot \sin(\alpha ) \end{array}$$

A swirl vector field can be modeled using three scalar parameters, b∈R, $x_{0}\in R$ and $y_{0}\in R$, as:

(3) $$\begin{array}{cc} p_{x} & =b\cdot \frac{y-y_{0}}{\sqrt{{\left(x-x_{0}\right)}^{2}+{\left(y-y_{0}\right)}^{2}}}\\ p_{y} & =b\cdot \frac{-x+x_{0}}{\sqrt{{\left(x-x_{0}\right)}^{2}+{\left(y-y_{0}\right)}^{2}}} \end{array}$$

where $x_{0}$ and $y_{0}$ control the location of the vortex of the swirl, the absolute value of *b* controls the strength of the swirl and the sign of *b* controls whether the swirl rotates clock or counterclockwise.

In this work, for simplicity, we consider only horizontal currents, which we model using three scalar parameters, c∈R, d∈R and $y_{0}\in R$, as follows:

(4) $$\begin{array}{cc} p_{x} & =c\cdot e^{-\frac{{\left(y-y_{0}\right)}^{2}}{d^{2}}}\\ p_{y} & =0 \end{array}$$

where $y_{0}$ locates the maximum current strength *y*-coordinate, the absolute value of *d* indicates the breadth of the current, the absolute value of *c* controls the strength of the current and the sign of *c* controls whether the current direction is positive or negative.

## 3. Optimal Control Motion Planning

An Optimal Control Problem (OCP) is an extension of the optimization problem to the case in which time is involved, and we want to obtain a trajectory that is optimal in a certain sense. In our case, a typical OCP is to determine the control trajectory (i.e., the value of the acceleration as a function of time) that minimizes the time to reach a certain position goal. There are several tools that could be used to solve such an OCP, depending on whether the time is discrete or continuous, and the literature on the topic is extensive (see [2,25,26,27] and their references). In this work, we will work using continuoustime OCPs.

### 3.1. Continuous Time Optimal Control

Let us assume the following OCP formulation [2] p. 104:

(5) $$\begin{array}{cc} \min J\left(s(t),u(t)\right) & =K(\left(s_{t_{f}}\right),t_{f})+\int_{0}^{t_{f}}L\left(s(t),u(t),t\right)dt\\ s.t.\; & \frac{ds}{dt}=f\left(s(t),u(t),t\right)\\ \; & s\left(0\right)=s_{0}\\ \; & \Psi (s\left(t_{f}\right),t_{f})=0\\ \; & u\left(t\right)\in U,\;\forall t\in \left[t_{0},t_{f}\right] \end{array}$$

where we have that:

- $t\in [0,t_{f}],\;t\in R$ is the time, where $t_{f}$ is the final time;
- $s\left(t\right)\in R^{m}$ is the state trajectory, where s(t) is the state at time *t*;
- $u\left(t\right)\in R^{l}$ is the control trajectory, where u(t) is the control at time *t*. The controls belong to the set of admissible controls *U*;
- $J:R^{m}\times R^{l}\times R\to R$ is the cost functional to be minimized, that is formed by a terminal cost functional $K:R^{m}\times R\to R$ and a running cost functional $L:R^{m}\times R^{l}\times R\to R$;
- The transition function $f:R^{m}\times R^{l}\times R\to R^{m}$ controls the state evolution as a function of *t*, s(t) and u(t);
- $s_{0}\in R^{m}$ is the initial state;
- $\Psi :R^{m}\times R\to R^{r}$ is the final condition that must hold at $t_{f}$.

Since we consider that the transition function *f* is deterministic, if we have the initial conditions and a control trajectory, we can obtain the state trajectory and the total cost by integrating (5). Note that we consider that time is a continuous variable, thus the name of continuous time OCP; it is also possible to work using a discrete time OCP, where some important changes from (5) are that integrals are replaced by sums, and differential equations by difference equations, as shown in [2].

#### 3.1.1. Dynamic Programming Methods for Continuous Time

There are two classical approaches to solve the OCP (5): the minimum principle and dynamic programming methods [2], where the former provides necessary conditions and the latter, sufficient conditions. We use a dynamic programming approach, which is based on the concept of value function V(s,t), which is a continuous function that returns the optimal cost that can be obtained in state *s* at time *t*. The function *V* is obtained by solving a nonlinear first order Partial Derivative Equation (PDE) as Theorem 1 states, Section 3.3.5 in [2]:

**Theorem** **1** (Dynamic Programming: HJB)**.** *The sufficient conditions that a continuous and unique function V(s,t) has to satisfy in order to be the optimal solution to the control problem *(5)* are the following two expressions, known as the Hamilton–Jacobi–Bellman (HJB) equation:*

(6) $$\begin{array}{cc} 0 & =\frac{\partial V}{\partial t}+\min_{u\in U}\left[{\frac{\partial V}{\partial s}}^{T}f\left(s,u,t\right)+L\left(s,u,t\right)\right]\\ V & \left(s\left(t_{f}\right),t_{f})=K(s\left(t_{f}\right),t_{f})+\nu^{T}\Psi (s\left(t_{f}\right),t_{f}\right) \end{array}$$

Unfortunately, solving (5) using the HJB Equation (6) is, in many cases, intractable (a notable exception being the linear quadratic case, which has a closed solution based on the Ricatti equations, Section 3.5 in [2]). First, note that (6) involves solving a nonlinear PDE due to the minimization operator which need not have a classic solution, i.e., an everywhere continuously differentiable *V* function. As noted by [28], we may think of admitting weaker solutions, that is, *V* functions that are continuous but not everywhere differentiable.

Based on this idea, Crandall and Lions proposed the viscosity solutions [29]. The key idea is that a viscosity solution to the HJB equation is a continuous but not everywhere differentiable *V* function. The derivative of *V* at points in which the viscosity solution is not differentiable is the derivative of a smooth function which touches *V*. Some important characteristics of the viscosity solutions are [28]: (1) the viscosity method allows selecting a single weak solution and (2) the optimal value function is the single viscosity solution to the HJB equation. Hence, finding the viscosity solution to the HJB equation allows obtaining *V*, and thus, it is no surprise that a large research has been addressed to these viscosity solutions, as [28,29,30,31,32,33,34,35], to mention some.

Another key advance consists of using numerical approximations that are based on discretizing the state space that, in the limit, converge to the viscosity solution of the HJB [28]. The seminal paper in this area is [36], where the authors conclude that any approximation method that satisfies the properties of monotonicity, stability and consistency is guaranteed to converge in the limit to the viscosity solution. A popular approach that is based on this framework is the finite-difference upwind method [3,4], which is based on the following ideas:

- The state space is discretized. Note that this means that we may face the curse of dimensionality if *n* is large;
- The value function is estimated iteratively by approximating the derivative with respect to the state by using finite differences;
- Depending on the state drift, for each state, the derivative is approximated using the backwards or the forward finite difference approximation.

This method is shown to satisfy the three properties of monotonicity, stability and consistency in [5], and hence, it converges to the viscosity solution in the limit. Two useful sources to implement these methods are [4], where several schemes and examples are described, and [5], where the upwind scheme algorithm is thoroughly described. The main problem that the upwind method faces is that it suffers from the curse of dimensionality, and hence this method is generally not useful to solve problems with a state dimension higher than 3, as the problem (1).

There are several other methods proposed to solve the HJB equation in Theorem 1, such as the ones based on level-set method and semi-Lagrangian schemes [6], which, however, are also based on a discrete grid and, hence, subject to the curse of dimensionality. Several methods have been proposed to alleviate this problem and scale to large state spaces, such as [7,8], which require obtaining mathematical expressions that link the original state space with a reduced state space, where the HJB equation is solved. Finally, a very promising method makes use of the recent deep learning advances in order to solve the HJB approximately without being subject to the curse of dimensionality: the method is known as deep Galerkin method (DGM) [9,10], which we now proceed to explain.

#### 3.1.2. Deep Galerkin Method

Let us first assume that we know the optimal control $u^{*}$ that minimizes the second term of the first equation from (6). Hence, in this case, the PDE we have to solve is:

(7) $$\begin{array}{cc} 0 & =\frac{\partial V}{\partial t}+{\frac{\partial V}{\partial s}}^{T}f\left(s,u^{*},t\right)+L\left(s,u^{*},t\right)\\ V(s\left(t_{f}\right),t_{f}) & =K(s\left(t_{f}\right),t_{f})+\nu^{T}\Psi (s\left(t_{f}\right),t_{f}) \end{array}$$

DGM approximates V(s,t) using a deep neural network (DNN), whose inputs are the state vector *s* and the scalar *t*. By means of using the backpropagation algorithm, it is possible to obtain the exact gradients $\frac{\partial V}{\partial s}$ and $\frac{\partial V}{\partial t}$. The DGM takes advantage of this to approximate the PDE using batches of samples: given the state and time space, we sample two sets of points: a set of interior points (s,t) that belong to the interior of the state and time space and a set of terminal points $\left(s,t_{f}\right)$. The set of interior points is used to minimize the following training loss:

(8) $${\left(\frac{\partial V}{\partial t}+{\frac{\partial V}{\partial s}}^{T}f\left(s,u^{*},t\right)+L\left(s,u^{*},t\right)\right)}^{2}$$

where we note that (8) is the squared error of the first term from (7): as (8) approaches 0, the first term of (7) is approximated. Then, the set of terminal points is used to train the NN in order to fulfil the final condition from (7) as:

(9) $${\left(V(s\left(t_{f}\right),t_{f})-K(s\left(t_{f}\right),t_{f})+\nu^{T}\Psi (s\left(t_{f}\right),t_{f})\right)}^{2}$$

where again, as (9) approaches 0, the final condition from (7) is approximated. Thus, the DGM approximately provides a solution for (7) by minimizing the sum of the loss terms (8) and (9). As the loss sum approaches 0, the DNN is the approximation of the optimal value function. Note that a very important property of the DGM is that is a meshless method: we do not train the DNN using a mesh over the state space, but samples, and let the DNN generalize to the states that have never been seen before. As shown by [9], the DGM is able to solve PDEs in very high dimensional spaces without being subject to the curse of dimensionality; due to this significant advantage, we used the DGM in this work.

However, the original DGM method, proposed in [9], assumed that the optimal control $u^{*}$ is known, which need not be the case in all control problems. In order to overcome this problem, ref. [10] proposed a modification of the DGM known as DGM-PI (Deep Galerkin Method—Policy Iteration), in which a second DNN is used to approximate the optimal policy function. In our problem, however, we do know the optimal control $u^{*}$, so we can use the standard DGM.

### 3.2. Continuous Time Surrogate Control

The continuous time OCP that we want to solve is the problem of minimizing the total time that it takes to an AUV to reach the origin. Hence, in our OCP, K=0, L=1, the final position must be the origin, the transition model is (1) and the control is the acceleration angle; thus, the OCP we want to solve is:

(10) $$\begin{array}{cc} \min J & =t_{f}\\ s.t.\; & \dot{x}\left(t\right)=v_{x}\left(t\right),\;\dot{y}\left(t\right)=v_{y}\left(t\right)\\ \; & \dot{v_{x}}\left(t\right)=\cos\left(\theta \left(t\right)\right)+p_{x}\left(x\left(t\right),y\left(t\right)\right)-k_{f}\cdot v_{x}\left(t\right)\\ \; & \dot{v_{y}}\left(t\right)=\sin\left(\theta \left(t\right)\right)+p_{y}\left(x\left(t\right),y\left(t\right)\right)-k_{f}\cdot v_{y}\left(t\right)\\ \; & s\left(0\right)=\left(x\left(0\right),y\left(0\right),v_{x}\left(0\right),v_{y}\left(0\right)\right)\\ \; & x\left(t_{f}\right)=y\left(t_{f}\right)=0,\;\theta \left(t\right)\in \left[0,2\cdot \pi \right),\;\forall t\in [0,t_{f}] \end{array}$$

and the HJB Equation (6) for this OCP is:

(11) $$\begin{array}{cc} 0 & =\frac{\partial V}{\partial t}+\min_{\theta }[\frac{\partial V}{\partial x}\cdot v_{x}\left(t\right)+\frac{\partial V}{\partial y}\cdot v_{y}\left(t\right)\\ \; & +\frac{\partial V}{\partial v_{x}}\cdot (\cos\left(\theta \right)+p_{x}\left(x\left(t\right),y\left(t\right)\right)-k_{f}\cdot v_{x}\left(t\right))\\ \; & +\frac{\partial V}{\partial v_{y}}\cdot (\sin\left(\theta \right)+p_{y}\left(x\left(t\right),y\left(t\right)\right)-k_{f}\cdot v_{y}\left(t\right))+1]\\ V & (x,y,v_{x},v_{y},t_{f})=0 \end{array}$$

### 3.3. Surrogate OCP for Continuous Time

However, we cannot solve (10) using DGM directly, as DGM assumes a fixed final time $t_{f}$, and in (10), $t_{f}$ is the minimization objective, and hence it is not fixed but free. Instead, we propose the following surrogate OCP

(12) $$\begin{array}{cc} \min J & =\int_{0}^{t_{f}}\tanh\left(\sqrt{x{\left(t\right)}^{2}+y{\left(t\right)}^{2}}\right)dt\\ s.t.\; & \dot{x}\left(t\right)=v_{x}\left(t\right),\;\dot{y}\left(t\right)=v_{y}\left(t\right)\\ \; & \dot{v_{x}}\left(t\right)=\cos\left(\theta \left(t\right)\right)+p_{x}\left(x\left(t\right),y\left(t\right)\right)-k_{f}\cdot v_{x}\left(t\right)\\ \; & \dot{v_{y}}\left(t\right)=\sin\left(\theta \left(t\right)\right)+p_{y}\left(x\left(t\right),y\left(t\right)\right)-k_{f}\cdot v_{y}\left(t\right)\\ \; & s\left(0\right)=\left(x\left(0\right),y\left(0\right),v_{x}\left(0\right),v_{y}\left(0\right)\right)\\ \; & \theta \left(t\right)\in \left[0,2\pi \right),\;\forall t\in \left[0,t_{f}\right] \end{array}$$

where the main changes are the following ones:

- The problem now has a fixed terminal time $t_{f}$, which is required by the DGM;
- As $t_{f}$ is fixed, we must change the functional *J*. Recall that our target was to move the AUV as close as possible to the origin; we achieve this by using, as a running cost, a hyperbolic tangent function that depends on the distance of the AUV to the origin at each time, which is $\sqrt{x{\left(t\right)}^{2}+y{\left(t\right)}^{2}}$. Thus, note that L→1 when the AUV is far from the origin, and as the AUV approaches the origin, L→−1. In other words, our surrogate cost functional penalizes for being far from the origin, and rewards positions of the AUV that are as close as possible to the origin.

Thus, the HJB Equation (6) for the surrogated OCP is:

(13) $$\begin{array}{cc} 0 & =\frac{\partial V}{\partial t}+\min_{\theta }[\frac{\partial V}{\partial x}\cdot v_{x}\left(t\right)+\frac{\partial V}{\partial y}\cdot v_{y}\left(t\right)\\ \; & +\frac{\partial V}{\partial v_{x}}\cdot (\cos\left(\theta \right)+p_{x}\left(x\left(t\right),y\left(t\right)\right)-k_{f}\cdot v_{x}\left(t\right))\\ \; & +\frac{\partial V}{\partial v_{y}}\cdot (\sin\left(\theta \right)+p_{y}\left(x\left(t\right),y\left(t\right)\right)-k_{f}\cdot v_{y}\left(t\right))\\ \; & +\tanh\left(\sqrt{x{\left(t\right)}^{2}+y{\left(t\right)}^{2}}\right)]\\ V & \left(x,y,v_{x},v_{y},t_{f}\right)=0 \end{array}$$

As we have mentioned in Section 3.1.2, in order to use DGM to solve (13), we need to solve the minimization problem in the PDE. Note that the minimization problem is the following, which we obtain by dropping all terms that do not depend on θ

(14) $$\min_{\theta }\left[\frac{\partial V}{\partial v_{x}}\cdot \cos\left(\theta \right)+\frac{\partial V}{\partial v_{y}}\cdot \sin\left(\theta \right)\right]$$

By obtaining the derivative of (14) and equalling to zero, we obtain the candidate points to be a minimum:

(15) $$\begin{array}{c} \theta_{DGM}^{*}=\left\{\arctan\left(\frac{\frac{\partial V}{\partial v_{x}}}{\frac{\partial V}{\partial v_{y}}}\right),\arctan\left(\frac{-\frac{\partial V}{\partial v_{x}}}{-\frac{\partial V}{\partial v_{y}}}\right)\right\} \end{array}$$

where the sign of each fraction determine the quadrant of the angle. Note that there are two candidate angles: the first one corresponds to the minimization solution, in which the AUV travels towards the origin, and the second corresponds to the maximization solution, in which the AUV tries to separate from the origin. Also, observe that we constrain the controls to lie in the set θ∈[0,2π) (as shown in (10) and (12)), and this constrain is naturally enforced by using the arctan operation. Thus, we now can solve the surrogate OCP (12), by making use of DGM to approximate the value function that satisfies the HJB PDE (13).

## 4. Empirical Simulation

In this section, we extensively study the results obtained by making use of the OCP tools explained in Section 3 applied to the setup described in Section 2. The steps we follow are:

- First, we explain the setup we use for our simulations in Section 4.1;
- Then, we train DGM and obtain the optimal control for the surrogate problem. We study the value function and control functions obtained, as well as the training convergence, in Section 4.2;
- Afterwards, we study the performance of DGM in the surrogate problem (12) in Section 4.3;
- Finally, we intensively study how the control obtained in the surrogate problem applies to our original problem, i.e., the time minimization problem (10), in Section 4.4.

### 4.1. Simulation Setup

As mentioned, we validate our ideas by solving the surrogate OCP (12) using the DGM. For all our simulations, we use the following discretized version of (1):

(16) $$\begin{array}{cc} x_{n+1} & =x_{n}+\Delta \cdot v_{x,n}\\ y_{n+1} & =y_{n}+\Delta \cdot v_{y,n}\\ v_{x,n+1} & =v_{x,n}+\Delta \cdot (\cos\left(\theta_{n}\right)+p_{x}\left(x_{n},y_{n}\right)-k_{f}\cdot v_{x,n})\\ v_{y,n+1} & =v_{y,n}+\Delta \cdot (\sin\left(\theta_{n}\right)+p_{y}\left(x_{n},y_{n}\right)-k_{f}\cdot v_{y,n}) \end{array}$$

where *n* denotes time indexes, Δ=0.01 s is the time step, and we set the friction parameter $k_{f}=0.5$. Note that we solve the OCP using continuous time tools, but we simulate using a discrete time setup: for values small enough of Δ, the error introduced by the discretization will be negligible.

As disturbances, we used the three models introduced in Section 2.2: a constant field disturbance following (2), with parameters (a,α)=(1/2,π/4); a swirl following (3), with parameters $\left(b,x_{0},y_{0}\right)=\left(1/2,5,1\right)$; and a current following (4), with parameters $\left(c,y_{0},d\right)=\left(1,5,3\right)$.

Finally, we compared the results obtained by DGM with the following baseline control $\theta_{b}\left(t\right)$, which consists of always accelerating towards the origin:

(17) $$\theta_{b}\left(t\right)=\arctan\left(\frac{-y(t)}{-x(t)}\right)$$

Note that the baseline is an intuitive control, which does not take into account the velocities nor the disturbances. Yet, if the velocities have a small magnitude and the the disturbances have a small effect, this baseline will provide good results, which we try to improve by using the DGM.

### 4.2. Training Results

**Figure 1:**
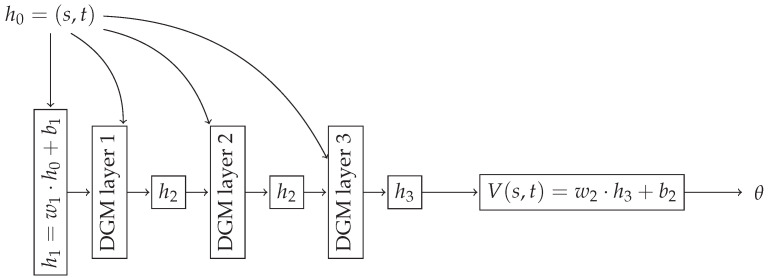
Block diagram showing the DGM architecture used in this work. Each DGM layer follows the implementation detailed in [10], where $w_{i}$ and $b_{i}$ denote weights and biases of the initial and final feed-forward layers. Each DGM layer is similar to an LSTM layer, as detailed in [10], and we used a dimension of 50 nodes for each of the 3 DGM layers. Note that the input is the pair (s,t) and the output of the neural network is the value function V(s,t). We obtained the control θ using (15). After convergence, both the value function and the control approximated the optimal ones.

We proceeded to train the DGM to solve the OCP (12), that is, we used the DGM to solve the HJB (13). We followed the DGM implementation used in [10]. For each of the three disturbances we studied, we used a three layer DGM neural network, where each layer has 50 nodes. The architecture can be seen in Figure 1. We trained the DGM during $10^{4}$ epochs; in each epoch, we randomly sampled $10^{4}$ interior points to minimize the loss (8) and other $10^{4}$ terminal points to minimize (9). Then, these points were used to minimize both losses during 10 iterations, and then, we started another epoch, where we sampled new points. As optimization algorithm, we used Adam [37], as it is a widely used minimization algorithm to train neural networks. Each time that we sampled state points, we followed a uniform distribution where x(t),y(t)∈[−10,10] m, and $v_{x}\left(t\right),v_{y}\left(t\right)\in \left[-2,2\right]$ m/s. Finally, note that we set $t_{f}=10$ s.

**Figure 2:**
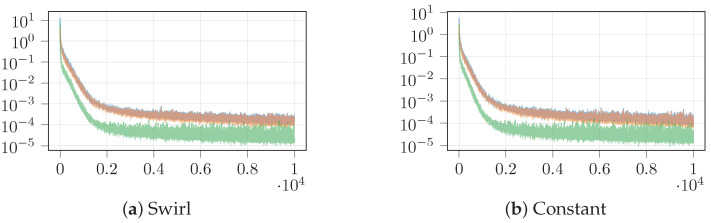

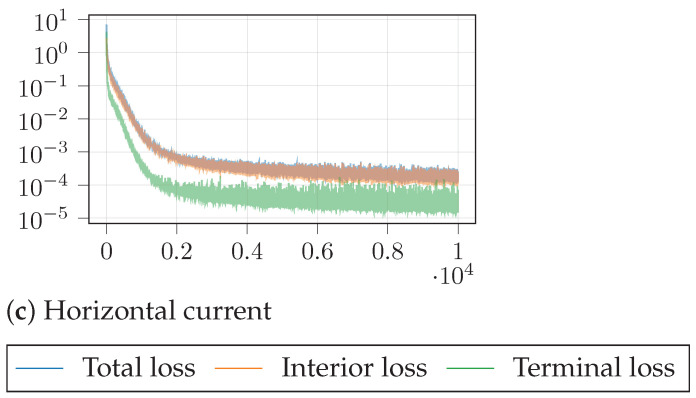
Training losses of DGM for the surrogated OCP solution (13): the horizontal axis represents the training epoch, while the vertical axis represents the losses (lower is better). We represent the total loss, which is the sum of the interior loss (8) and the terminal loss (9): as the latter is around one order of magnitude smaller than the former, the total loss is dominated by the interior loss. Note how all losses are minimized as the training advances, which means that DGM converges to a solution of the HJB equation.

**Figure 3:**
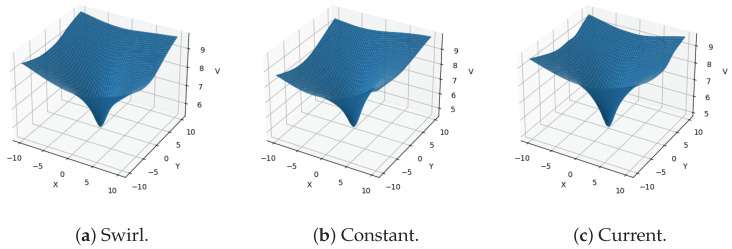
Value function obtained by DGM for each disturbance, where the horizontal axes represent *x* and *y*, respectively, and considering that $v_{x}=v_{y}=0$. The value plot represent the value function V(s,t) that DGM estimates as the solution to the HJB Equation (13) at t=0; note that each disturbance model yields a different value function, where we can see the shape of the disturbance (compare with the disturbances shown in Figure 5).

**Figure 4:**
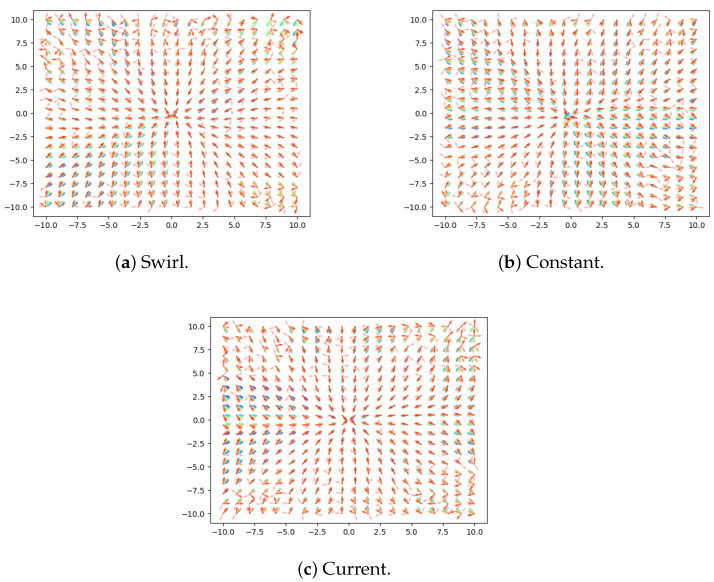
Control obtained by DGM for each disturbance, where the horizontal and vertical axes represent *x* and *y*, respectively, and considering that $v_{x}=v_{y}=0$. Note that these plots represent the optimal control obtained using (15), which depends on the gradient of the value function from Figure 3. We represent the control for different values of *t*, where cold colors represent t→0 and warm colors $t\to t_{f}$. We observe that the quality of the control obtained by DGM worsens slightly as the distance from the origin increases, as mentioned.

**Figure 5:**
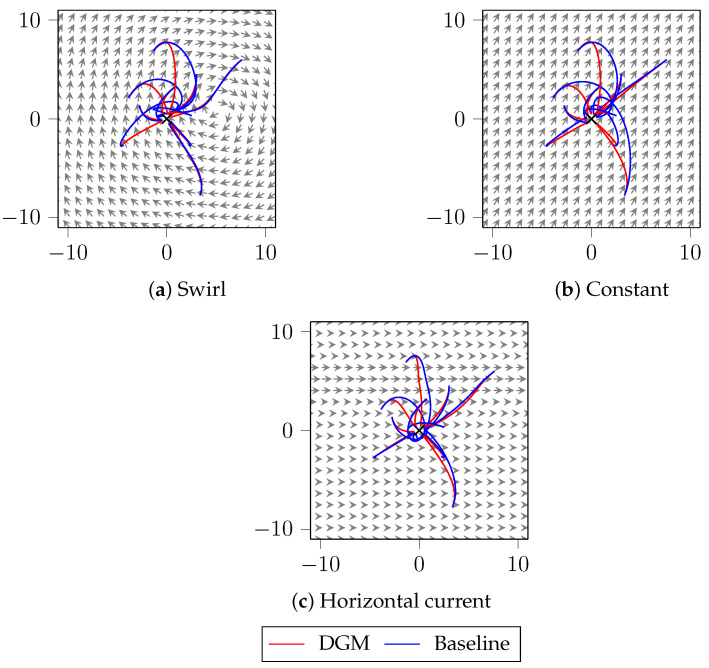
Example trajectories for each control law and disturbance, where the horizontal axis denotes the *x* coordinate, and the vertical axis is *y*. The vector field represents the disturbance, the red trajectories are obtained using DGM and the blue trajectories are obtained with the baseline control (17). Observe how DGM trajectories differ the most as the disturbance effect increases, thus giving a strong advantage to DGM over the baseline when the disturbance effect is not negligible.

With these parameters, DGM converges, as shown in Figure 2, where we can see that both losses are minimized during training and reach low values. This means that we obtained a neural network that estimates both the value function V(s,t) and the optimal control $\theta^{*}\left(t\right)$, and both can be observed in Figure 3 and Figure 4 for the concrete cases when $v_{x}=v_{y}=0$. First, in Figure 3, we observed the value function as a function of $\left(x,y,v_{x}=0,v_{y}=0,t=0\right)$, where we note that we have to particularize the values of $v_{x}$, $v_{y}$ and *t* to obtain a 3-D plot. As expected, each value function is different, reflecting the influence of the disturbance. For instance, by comparing the value function of the constant disturbance in Figure 3 and the disturbance effect from Figure 5, we can observe how the cost is smaller as *x* and *y* decrease, as the disturbance pushes the AUV towards the origin, while the cost increases as *x* and *y* increase, as the disturbance pushes the AUV far from the origin.

Figure 4 shows the optimal control obtained using the DGM, where the control is obtained by following (15). Note that the optimal control depends on the derivative of the value function; hence, it may suffer from noise if the value function estimate is not good. We observe several interesting points in this figure: First, note how the optimal control obtained is very similar to the baseline control (17), as the control tends to be pointing towards the origin. Second, note how there are variations in the control depending on the disturbance; this is expected, as the DGM is able to obtain optimal control functions for each concrete disturbance. Finally, note that when we are far from the origin, the quality of the control obtained by DGM worsens, as it may indicate to accelerate in directions that separate the AUV from the origin. This is due to the fact that the cost functional *J* in (12) depends on the distance to the origin, and note that we sample uniformly in *x* and *y*, which in turn means that the distribution of points used to train DGM is not constant with the distance, as lower distances will have more data. Hence, what we observe is reasonable: larger distances present worse estimations, as they have appeared less frequently during training.

### 4.3. Surrogate Problem Results

Now, we proceed to compare the results obtained by the control obtained by the DGM and the baseline control. In order to benchmark the performance of both controls, we set 100 initial states $s_{k}\left(0\right)$, where k∈{1,2,3,⋯,100}, where each initial state is formed by an initial velocity $v_{x}\left(0\right),v_{y}\left(0\right)\in \left[-2,\;2\right]$ m/s, and an initial position x(0),y(0)∈[−8,8] m. Note that the initial velocity is sampled in the same limits that were used to train the DGM, but the initial position is sampled using reduced limits to avoid the points in which DGM obtained a bad estimation of the control, as explained in the previous section.

We define the cost error as follows:

(18) $$e=\sum_{k=1}^{100}e_{k}=\sum_{k=1}^{100}\left(J\left(s_{k}\left(0\right),\theta_{DGM}\left(t\right)\right)-J\left(s_{k}\left(0\right),\theta_{b}\left(t\right)\right)\right)$$

where *e* is the error between the total costs of the DGM control $\theta_{DGM}\left(t\right)$ and the baseline control $\theta_{b}\left(t\right)$, averaged using the 100 different initial states $s_{k}\left(0\right)$. Note that as we are dealing with a minimization problem, e≤0 means that the DGM obtains better results than the baseline (i.e., a lower cost—that is, the DGM is better), and e>0 means that the baseline returns a trajectory with smaller cost (i.e., the baseline is better). Note that we use the cost functional *J* from (12).

The results obtained can be seen in Table 1, where we represent both the error *e* following (18) and the proportion of trajectories in which the DGM provides a lower cost than the baseline, i.e., the improvement proportion of trajectories. Note that in both metrics, the DGM provides considerably better results than our baseline: the error is always negative, which means that the DGM is better than the baseline, and then, the improvement proportion of DGM is higher than 80% across all disturbances. Note that this is an important result, as we have trained the same architecture of DGM and it has obtained good results for all the disturbances tested; this implies that the DGM may generalize and work with many other types of disturbances, which makes it a very powerful and flexible approach.

### 4.4. Original Problem Results

The results from the previous section were expected, as we trained the DGM to approximate the solution of the HJB (13). Now, it is time to evaluate whether our surrogate problem is effective in solving our original OCP (10). In order to evaluate this, we used the same 100 initial conditions from the previous section to evaluate the cost that yield both the baseline and DGM controls. Note that the key difference with the previous section is that now, instead of evaluating the cost using the cost functional from (12), we use the cost functional *J* from (10). Additionally, the original problem finished when the origin was reached; in our case, as we implemented the discrete version (16), we were satisfied that the origin was reached when the distance to the origin was smaller than 0.5 m.

The results obtained are in Table 2 and Figure 5. First, in Table 2, we represent both the error *e* following (18) and the proportion of trajectories in which the DGM provides a lower cost than the baseline, i.e., the improvement proportion of trajectories. Note that, in both metrics, the DGM provides considerably better results than the baseline: first, the error is always negative, which means that the DGM is better than the baseline, and then, the improvement proportion of DGM is of 87% for all disturbances tested.

Finally, in Figure 5, we plot several trajectories for each disturbance and control law. Observe that the subtle differences in controls between the baseline (17) and the control obtained by DGM (see Figure 4) are translated into significant differences in terms of trajectories, and hence, in costs. DGM is able to adapt to the disturbance, and specially in regions where the effect of the disturbance is strong, as close to the swirl or current centers, its advantage over the baseline is clearly seen, as it is able to obtain a control law that minimizes the time to reach the origin. As we mentioned, in regions where the disturbance effect is small, the baseline control is good, and the DGM provides similar values to it.

## 5. Conclusions

In this work, we propose solving the OCP for navigation by means of using the DGM, an efficient meshless method that allows approximating the solution of the HJB equation making use of the recent advances in deep learning in an efficient way. Since optimal navigation problems generally are formulated in terms of minimizing the time in which a certain target is reached, we propose a novel approach in which we use a surrogate problem to transform this problem into a fixed-horizon problem, which we can solve using the DGM. When we test in the presence of disturbances, we note that DGM offers strong results compared to the baseline consisting in accelerating towards the origin, as it provides significant improvements in terms of cost, specially when the effect of the disturbances is not negligible.

There are several future lines of work that may arise from this work. First, it would be interesting checking how the DGM behaves when using different disturbances. Even though in this work we have tested using three different disturbance models, which are swirls, currents and constant disturbances, it would be interesting checking whether the DGM works on a wider set of disturbances and disturbances parameters. Another line of work could be trying to address the border effects that appeared in the control in Figure 4; one possibility would be sampling during training using a uniform distribution taken from Polar coordinates rather than Cartesian, so that the DGM is trained uniformly on the distances to the origin. Another possible line would consist of evaluating the effects of the uncertainties in localization that arise in the underwater medium [23] in our proposed method.

Another line of work would consist of adding more constrains to the problem we are solving, to make it more realistic in different aspects. One of them could be using a noisy observation as input, instead of the actual state, as underwater location mechanisms are noisy. Another would be adding constrains to the control that reflect the actuators limitations. Note that (5) can deal with admissible controls, and in (10) we limit our controls to lie in the set θ∈[0,2π) (which is enforced by the arctan operation in (15)). However, different control constrains could be enforced in our formulation depending on the AUV actuators. Additionally, our approach could also be tested using obstacles, by means of modifying the cost functional to have an additional term that increases the cost as the distance to an obstacle increases.

It would also be possible to add uncertainty about the disturbance. Note that, in this work, we have trained a neural network for each disturbance, and hence, our method requires knowing the disturbance in advance, as well as its parameters. A possible way to address this would be to extend the state to include the disturbance parameters as inputs: the negative counterpart would be an increase in the computational load as a result of the growth of the state space.

Finally, it would also be interesting checking the performance of the DGM compared to other methods used to approximately solve OCPs, such as deep reinforcement learning ones, which have already been applied to navigation ([38,39]) and offer an interesting alternative to compare the DGM with.

## Figures and Tables

**Table 1:** Results for the surrogate OCP (12), comparing DGM and the baseline control. DGM provides better results, not only in terms of lower cost and average error (remember that negative error values mean that DGM is better), but also in that more than 80% of the trajectories present a lower cost with DGM.

Disturbance	Swirl	Current	Constant
Average *J* DGM	5.26	4.26	5.12
Average *J* baseline	6.97	4.64	6.95
*e*	−1.70	−0.38	−1.83
Improvement proportion	0.84	0.81	0.83

**Table 2:** Results for the original OCP (10), comparing DGM and the baseline control. The DGM provides better results, not only in terms of lower cost and average error (remember that negative error values mean that the DGM is better), but also in that more than 85% of the trajectories present a lower cost with the DGM.

Disturbance	Swirl	Current	Constant
Average *J* DGM	5.45	4.75	5.63
Average *J* baseline	7.68	5.55	7.81
*e*	−2.25	−0.80	−2.17
Improvement proportion	0.87	0.87	0.87

## Data Availability

Data sharing is not applicable.
